# MicroRNA-30a-5p suppresses proliferation, invasion and tumor growth of hepatocellular cancer cells via targeting FOXA1

**DOI:** 10.3892/ol.2021.12835

**Published:** 2021-06-02

**Authors:** Shuliang Zhang, Qin Liu, Qi Zhang, Ling Liu

Oncol Lett 14: 5018-5026, 2017; DOI: 10.3892/ol.2017.6745

Following the publication of this paper, it was drawn to the Editors’ attention by a concerned reader that certain of the Transwell assay data shown in Fig. 2E and F and in Fig. 4E and F were strikingly similar to data appearing in different form in another article by different authors. Owing to the fact that the contentious data in the above article were already under consideration for publication elsewhere prior to its submission to *Oncology Letters*, the Editor has decided that this paper should be retracted from the Journal. After having been in contact with the authors, they agreed with the decision to retract the paper. The Editor apologizes to the readership for any inconvenience caused.

